# An Insight on Microfluidic Organ-on-a-Chip Models for PM_2.5_-Induced Pulmonary Complications

**DOI:** 10.1021/acsomega.3c10271

**Published:** 2024-03-07

**Authors:** Disha Shah, Bhavarth Dave, Mehul R. Chorawala, Bhupendra G. Prajapati, Sudarshan Singh, Gehan M. Elossaily, Mohd Nazam Ansari, Nemat Ali

**Affiliations:** † Department of Pharmacology and Pharmacy Practice, L. M. College of Pharmacy Navrangpura, Ahmedabad, Gujarat 380009, India; ‡ Office of Research Administration, Chiang Mai University, Chiang Mai 50200, Thailand; § Department of Pharmaceutical Sciences, Faculty of Pharmacy, Chiang Mai University, Chiang Mai 50200, Thailand; ∥ Department of Pharmaceutics and Pharmaceutical Technology, Shree S. K. Patel College of Pharmaceutical Education and Research, Ganpat University, Mehsana, Gujarat 384012, India; ⊥ Department of Basic Medical Sciences, College of Medicine, AlMaarefa University, P.O. Box 71666, Riyadh 11597, Saudi Arabia; # Department of Pharmacology and Toxicology, College of Pharmacy, Prince Sattam Bin Abdulaziz University, Alkharj 11942, Saudi Arabia; 7 Department of Pharmacology and Toxicology, College of Pharmacy, King Saud University, P.O. Box 2457, Riyadh 11451, Saudi Arabia

## Abstract

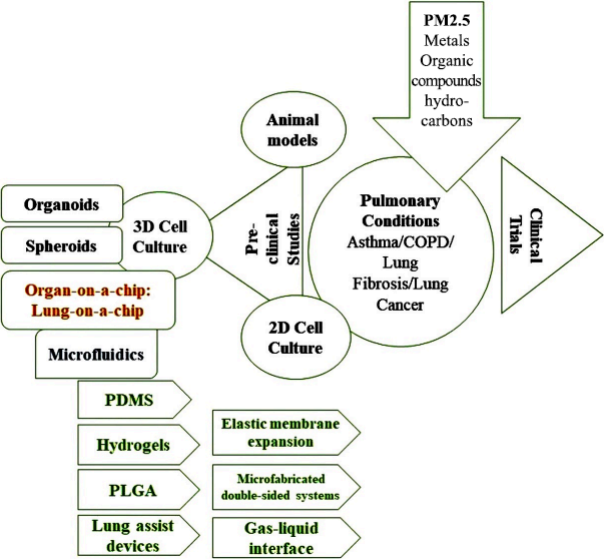

Pulmonary diseases like asthma, chronic obstructive pulmonary disorder, lung fibrosis, and lung cancer pose a significant burden to global human health. Many of these complications arise as a result of exposure to particulate matter (PM), which has been examined in several preclinical and clinical trials for its effect on several respiratory diseases. Particulate matter of size less than 2.5 μm (PM_2.5_) has been known to inflict unforeseen repercussions, although data from epidemiological studies to back this are pending. Conventionally utilized two-dimensional (2D) cell culture and preclinical animal models have provided insufficient benefits in emulating the in vivo physiological and pathological pulmonary conditions. Three-dimensional (3D) structural models, including organ-on-a-chip models, have experienced a developmental upsurge in recent times. Lung-on-a-chip models have the potential to simulate the specific features of the lungs. With the advancement of technology, an emerging and advanced technique termed microfluidic organ-on-a-chip has been developed with the aim of identifying the complexity of the respiratory cellular microenvironment of the body. In the present Review, the role of lung-on-a-chip modeling in reproducing pulmonary complications has been explored, with a specific emphasis on PM_2.5_-induced pulmonary complications.

## Introduction

Air pollution is the main environmental factor that plays a significant role in various illnesses. Its negative impact encompasses a range of health complications such as cerebrovascular disorders, pre-eclampsia, hyperactive disorders, bronchitis, emphysema, and neurodegenerative diseases.^1,2^ The primary cause of air pollution worldwide is the combination of harmful gases and particulate matter, which can severely affect human health. According to the World Health Organization (WHO), approximately 7 million people, mostly from low- or middle-income countries, lose their lives each year due to exposure to polluted air. In 2016, the recorded number of deaths attributable to air pollution was around 4 million^3,4^ Particulate matter (PM) is composed of a variety of particles, including nitrates, sulfates, endotoxins, and reactive particles such as iron, copper, nickel, etc., that harm the environment and overall bodily functions. PM is further subclassified based on particle size, which includes coarse PM_10_ (diameter of <10 μm), fine PM_2.5_ (diameter of <2.5 μm), and ultrafine PM_0.1_ (diameter of <0.1 μm).^5^ Investigations carried out across the globe have revealed the role of particulate matter in causing air pollution, and it has been the cause of severe morbidity and mortality.^6^ These studies have particularly focused on the impact of particulate matter on global health and air pollution rather than gaseous components. They observed that PM can influence the effects of pollution on human health in several ways and can highly affect the functioning of the cardiovascular system in humans regardless of the duration of exposure. Several cardiovascular abnormalities, such as ischemic heart disease, heart failure, thrombotic stroke, myocardial infarction, etc., have been known to occur following exposure to PM.^7^ Additionally, PM can also impair the functioning of the endocrine system, put the individual at risk for developing metabolic disorders such as diabetes mellitus (DM), and aggravate their risk for cardiovascular disorders.^8,9^ Novel epidemiological studies provide insights into the role of PM in air pollution. It is essential to understand the role of PM and the precise mechanisms through which it causes damage to human health.^5^

## PM_2.5_

The Global Burden of Disease 2015 (GBD 2015) puts the PM_2.5_ particulate inhalation as the fifth most leading cause of death due to respiratory illnesses around the globe.^4,10^ PM_2.5_ is a category of particles with a size lesser than or equal to 2.5 μm that can be inhaled during physiological respiration and enter the lungs. These particles affect the environment in several ways, and their source may either be natural or due to human activities.^11^ PM_2.5_ belongs to the category of fine particulate matter and possesses a size so small that it can reach easily to the respiratory system through an inhalational route and can further enter the lung alveoli, through which it makes its way into the systemic circulation and enters the bloodstream.^12,13^ Its exact composition includes inorganic ions, minerals, black carbon, polycyclic aromatic hydrocarbons, volatile organic hydrocarbons, etc., which constitute 70–80% of PM_2.5_ particles.^14^ They have the advantage of having a larger surface area due to their smaller size, as a result of which they spread more readily into the environment, travel long distances, and affect larger populations.^15^ The precise mechanisms of how PM_2.5_ causes respiratory illnesses are unclear; however, it is proposed that PM particles, through inhalation, are accumulated in the lung alveoli, wherein they cause the stimulation of alveolar receptors leading to the generation of an inflammatory response and the subsequent release of inflammatory mediators into the bloodstream. This ultimately causes an imbalance in the autonomic nervous system (ANS) and the neuroendocrine pathway.^16,17^

## PM_2.5_-Associated Pulmonary Infections

PM_2.5_ exposure increases the likelihood of lung infections, especially in young people, the elderly, and individuals having comorbid conditions.^18−20^ These particles also disrupt the host’s immunological defenses, leading to immunosuppression and making them highly susceptible to developing immune-related diseases and infections.^21^ According to epidemiologic studies, inhaling PM_2.5_ increases the risk of developing respiratory illnesses. The vulnerability to respiratory system infections may be attributed to the host defense failure brought on by PM_2.5_ exposure.^10^ This hypothesis has been backed by animal studies investigating the role of PM_2.5_ in causing respiratory and lung infections, which revealed that PM_2.5_ inhalation led to the development of lung infections within animals.^22,23^ Madsen and co-workers conducted a study in which they observed the microorganisms that were present in the particulate matter, which led to infection in the lung alveoli. They concluded that methicillin-resistant *Streptococcus aureus* (MRSA) and *S. aureus* were found in the analysis of PM_2.5_.^24^ Furthermore, PM_2.5_ particles are also associated with viral infections following respiratory illnesses such as COVID-19.^25^ This Review summarizes the various infections and complications caused by PM_2.5_ particles, along with their precise mechanisms and the role of pulmonary microfluidic organ-on-a-chip models for the evaluation of these disease conditions and therapeutic drug development.

## Mechanisms Inducing PM_2.5_-Related Complications

**Figure 1:**
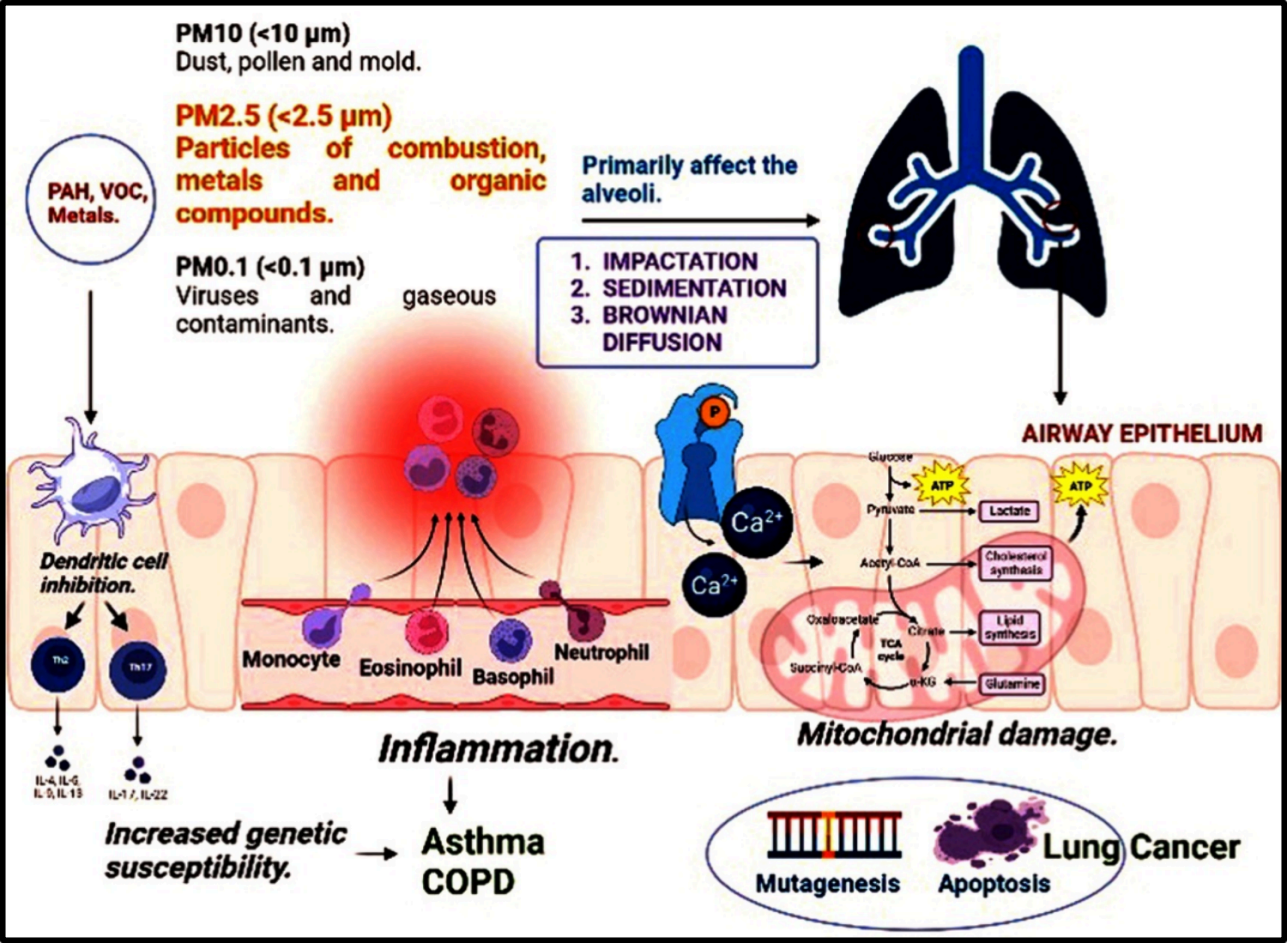
A brief schematic representation of the mechanism involved in PM_2.5_-induced pulmonary complications.

There are various mechanisms that lead to certain complications from PM_2.5_. In order to obtain definitive knowledge of how the complications occur, it is important to understand the mechanism through which they occur. Probable mechanisms put forward for causing pulmonary complications and infections are summarized herewith. Free radical production has been observed due to the metal and organic components of PM_2.5_, and the generation of toxic free radicals has been shown to cause damage within the lung cells. Also, replenishment or certain antioxidants has led to reduced damage, which shows the effects of ROS in lung injury.^26,27^ Examples of free radicals include hydroxyl ions (OH), and they are associated with exerting their harmful effects on doxyribonucleic acid (DNA). The various minerals and components of PM_2.5_ such as polycyclic aromatic hydrocarbons, aryl hydrocarbons, and lipopolysaccharides can produce these free radicals and cause their peroxidation, leading to the generation of Advanced lipid peroxidation end products (ALP).^28^ A study carried out by Mehta et al. concluded that these toxic free radicals can not only damage the DNA to be formed but can also mediate the replication of the damaged DNA, which leads to harmful reproduction of the damaged cell, promotes carcinogenesis and tumor formation in the lung, and causes lung cancer.^29^ Inhalation of PM_2.5_ triggers the inflammatory response, wherein inflammatory cytokines are released into the bloodstream, and this further activates overexpression of certain genes involved in inflammatory response control, ultimately leading to inflammatory cytokine injury.^30^ Sigaud et al. observed that neutrophils were released following exposure and inhalation of PM_2.5_ in humans.^31^ The various inflammatory markers released include interferon-ϒ (IFN-ϒ), interleukin (IL)-4, IL-10, IL-12, and IL-13, along with the recruitment of eosinophils.^32,33^ Disrupted calcium homeostasis is one of the significant mechanisms provoking disturbance due to PM_2.5_. As calcium is involved in the maintenance of normal cell physiology and functioning, increased levels can lead to stimulation of inflammation within the body and can cause damage to organs.^34,35^ The toxic free radicals produced due to the inhalation of PM_2.5_ lead to a subsequent increase in the levels of intracellular calcium (Ca^2+^) ions, which can further aggravate the peroxidation process and ROS formation.^35,36^ This could further lead to cell necrosis and delayed cellular apoptosis.^37^ An overview of the mechanisms leading to PM_2.5_-related pulmonary conditions is presented in Figure 1.

## Pulmonary Infections and Complications

PM_2.5_ particles are easily able to enter the lungs due to their fine size and larger surface area, which is the reason they leads to several pulmonary complications. As a result of various investigations conducted to highlight the pulmonary complications of PM_2.5_, it was observed that entry of PM_2.5_ into the lungs led to an exacerbation of the inflammatory response, which caused damage to the lung tissue both directly and through the aggravation of respiratory symptoms. The specific role of the generation of toxic free radicals and reactive oxygen species (ROS) was implicated in this.^26,38−40^ This may become more dangerous in the case of individuals already having a pre-existing respiratory condition and can further lead to further deterioration of their condition.^18,41,42^ Due to this, a variety of pulmonary complications, such as lung cancer, chronic obstructive pulmonary disease (COPD), asthma, etc., have been found to occur in the adult population and can result in reduced pulmonary function.^43−45^ The instances of respiratory infections occurring due to PM_2.5_ were assessed by analyzing hospital-related data, and it was confirmed that exposure to PM_2.5_ led to the development of respiratory infections in humans.^46^ More specifically, PM_2.5_ was positively correlated with upper respiratory tract infections (URTIs) such as rhinitis, laryngitis, tonsillitis, etc.^47,48^ It was noted that PM_2.5_ can also cause microbial infestation in the lungs in addition to tissue damage.^13^ These infections include H1N1 flu, severe acute respiratory syndrome (SARS), COVID-19, etc.^49,50^

## Conventional In Vitro Models to Understand the Effect of Particulate Matter

Cell culturing has developed into an essential tool for understanding the fundamental biophysical and biomolecular mechanisms by which cells create tissues and organs, how these tissues function, and how disease affects their functioning. When it comes to research tools for mimicking human development and a variety of disorders, in vitro cell cultures play a critical role. The existence, amount, or functional activity of a cell or tissue can be evaluated quantitatively by using cell culture assays. Biochemical research has used cell culture models as pioneers of basic research.^51^ Despite lacking the necessary tissue architecture, 2D cell cultures have a number of significant advantages over in vivo animal models. Although genetically modified mice play a crucial role in developmental cancer research, they are unable to accurately simulate the variety, physiology, and genetics of human diseases. When compared to animal experimentation, cell culture models offer significant advantages due to their inexpensive cost, short maintenance time, and great reproducibility. The applicability of cell culture models has increased through more easy functional analysis and simpler scaling. Comparing these high-throughput assays to costly, time-consuming, and labor-intensive animal models, they offer physiologically realistic models.^52^ Due to their simplicity, high productivity, reproducibility, and affordability, flat-support two-dimensional (2D) monocultures are the most widely used in vitro assay approaches to study the underlying cell behavior and discover a variety of biological and pharmacological applications. However, 3D cultures effectively harness the necessary cellular cross-talking networks with a superior ability to simulate in vivo settings compared to 2D cultures.^53^ Recent discoveries have shown a change in favor of 3D culture models because of the more precise biochemical and biomechanical microenvironments they offer. New research directions for examining the fundamental translational machinery, cell–cell interactions, cell–matrix interactions, and other cellular behaviors have been made possible by 3D models.^51^ The combination of 3D culture systems with regenerative medicine is likely to improve patient outcomes as existing technologies are further optimized and effective scaffolds are developed.^54^ These models present a physiologically suitable cellular environment, which offers significant potential for analysis of drug disposition and pharmacokinetics that affect medicine safety and efficacy from an early stage of drug development.^55^ These systems can take many different shapes, ranging from straightforward spheroids to sophisticated organoids and organs-on-chips. Other subcategories include static single-cell 3D models, cell coculture models with microfluidic control, and hybrid 3D systems.^56^ 3D models have proven to be superior platforms for cell- and organ-based experiments due to the limited applications and data extrapolation of 2D cell cultures. They make it possible to evaluate the accuracy of disease models made from cells taken from patients as well as to examine the safety of low-clearance medications and multiple dosing studies. To assess the impact of various drug administration routes on pharmacokinetics and to increase drug safety and efficacy, these organ-specific 3D models are implemented into a range of microphysiological systems.^56,57^ Spheroids are spherical arrangements of cells due to self-assembling aggregates that are formed due to integrins and extracellular matrix proteins. Although the latter are more frequently utilized, they can be developed utilizing a scaffold-based or scaffold-free technique. The cytoskeleton of the cells largely determines the mechanical integrity of spheroids. Spheroids are cell aggregates of one or more cells that are primarily produced from the original cells of an organ or tissue, but they do not architecturally resemble the organ or tissue under consideration, contrary to organoids.^58^ Organ-on-a-chip systems can better recreate complicated and important organs in vitro with the use of microfluidics technology, which captures the cellular milieu.^59,60^ An organoid is essentially a miniature organ created in a laboratory setting to imitate the biofunctional characteristics of the target organ.^61^ Another class of 3D culture scaffold-based models that is created from either adult stem cells or pluripotent stem cells going through spontaneous differentiation and self-organization is lung organoids. Ethical questions were raised when pluripotent stem cells (PSCs) were extracted from embryos during the blastocyst stage. However, they diminished after the induced pluripotent stem cell (iPSC) technology was developed. By increasing the expression of transcription factors linked to pluripotency, adult human fibroblasts can be isolated and transformed into pluripotent stem cells. Organoids created from iPSCs go through a self-autonomous process of organ development. The cells are instructed to develop into the histo-physiologically similar primitively organized organs.^62^ Organoids made from adult stem cells (ASCs) develop in a much more straightforward and long-lasting manner. ASCs, unlike iPSCs, do not need to be reprogrammed; instead, cells from the target organ are extracted from the subject, separated, and cultured to create a 3D architecture model with epithelial layers mimicking the target organ.^63^ With the development of 3D bioprinting, it is now possible to create complex organs and 3D tissue architectures in a scalable and reliable manner. Due to its effectiveness and reproducibility, this method has potential for the creation of appropriate and accurate disease models.^64^ Extracting biomaterials like cells, growth factors, etc., entails using 3D printing to mimic the native tissues.^65^ Cells, additives, and scaffolds make up bioink; however, microtissue- and autonomous self-assembly-based bioprinting provide scaffold-free methods.^66^ Although widely utilized for basic research puproses, these conventional in vitro techniques are associated with certain disadvantages. For an instance, the design and development of spheroids of uniform sizes is a lengthy and tedious procedure, and a very high shear force is required to maintain these cultures for longer durations.^67,68^ Organoids, on the other hand, face the limitations of requiring extracellular matrix and growth factor supplementation.^69^ Additionally, the use of matrigel for the generation of organoids is associated with significant animal use and exploitation.^70^ These animal-based origin scaffolds of matrigel also poses a risk of antigenic response.^71−74^ Despite the fact that 3D biopritning techniques provide high throughput analysis and improved cell viability, they face the issue of the generation of toxic degradation products, which are liable to cause immune responses.^75,76^ Moreover, designing bioinks with the optimal viscosity and biocompatibility is a difficult and expensive process.^77^ Additionally, there are several fallbacks associated with 2D models as compared to 3D cell culture methods. Two-dimensional models permit soluble elements to diffuse into the medium without creating a gradient. The development of a concentration gradient of soluble compounds like growth factors, on the other hand, is made possible by three-dimensional models, as demonstrated in several studies, notably in the context of microfluidic devices.^78−80^ Comparing 3D cell culture models to 2D models, which feature monolayers of cells, reveals a better picture of cell–cell interactions.^81−83^ Additionally, as 2D models lack the complexity necessary for cell growth, there is insufficient cell expansion. ASC proliferation has instead been found to be superior in 3D models.^84−86^

Cell lines grown in vitro are typically entirely inactive and devoid of physiological activity.^87^ This phenomenon also occurs in primary cultivated cells, and even when these activities are normal immediately after harvest it is very challenging to maintain cellular functions for extended periods of time. Conventional methods involve cultivating cells in a semistatic environment where experimental substances are only applied to the cells by diffusion.^88^ On the contrary, under in vivo conditions, cells acquire oxygen and nutrients via blood flow in addition to chemical stimulation and physical stimulation from the environment, such as stretching and shear stress. Such morphological and environmental variations between in vivo and in vitro conditions could be the cause of cellular function loss or deactivation in cultures.^89^

## Comparative Analysis of Conventional and Varied 3D Cell Culture Models

The introduction of 3D cell culture models has caused a paradigm change in the area of cell culture in recent years. We will explore a variety of 3D cell culture methods, emphasizing their special qualities and uses in biomedical research, such as animal models, 2D cell culture, spheroids, organoids, air–liquid interfaces, and microfluidic systems.^90^ For many years, the foundation of scientific research has been 2D cell cultures and traditional in vitro models. They frequently fail, however, to accurately capture the intricate physiological and pathological circumstances present in vivo. 3D cell culture models, on the other hand, seek to close this gap by offering a more biomimetic environment that enhances cellular interactions and creates structures that resemble tissues.^91^

Animal models are an integral component of biomedical research even though they are not the only method used in vitro. They offer a comprehensive understanding of physiological processes, but their widespread application is constrained by ethical issues, expensive prices, and species-specific variations.^92^ On the other hand, adding a 3D cell culture to animal models can improve the applicability and relevance of the findings. The conventional 2D cell culture is still commonly utilized because of its affordability and ease of use. However, its capacity to mimic intricate tissue architecture and function is constrained by the absence of spatial organization and cellular heterogeneity observed in vivo. The comparative analysis will evaluate the advantages and disadvantages of 2D models compared to 3D models.^93,94^

Spheroids, which enable cells to self-assemble into three-dimensional structures resembling natural tissues, mark a significant breakthrough in 3D cell culture. Spheroids are a desirable model for researching medication responses, tissue formation, and cancer biology because of their improved cell–cell interactions and nutrition and oxygen gradients.^94^ Organoids are self-organizing three-dimensional entities that resemble certain organs in both structure and function. They are created from stem cells or tissue fragments. This approach provides a great degree of complexity, making it possible to investigate the development of organs, mimic diseases, and apply personalized treatment.^95^ In an air–liquid interface culture, the basolateral side of the cells is kept in touch with the culture media while the apical surface of the cells is exposed to air. Because of its increased physiological relevance, this model is especially useful for researching respiratory epithelia, giving researchers a better understanding of medication absorption, toxicity testing, and airway illnesses.^96^ Microfluidic systems provide for fine control over the cellular microenvironment by integrating cells into small-scale devices. With the ability to create dynamic and adjustable settings, this technology can be used to research drug screening, disease modeling, and cell migration with more physiological relevance.^97^

**Figure 2:**
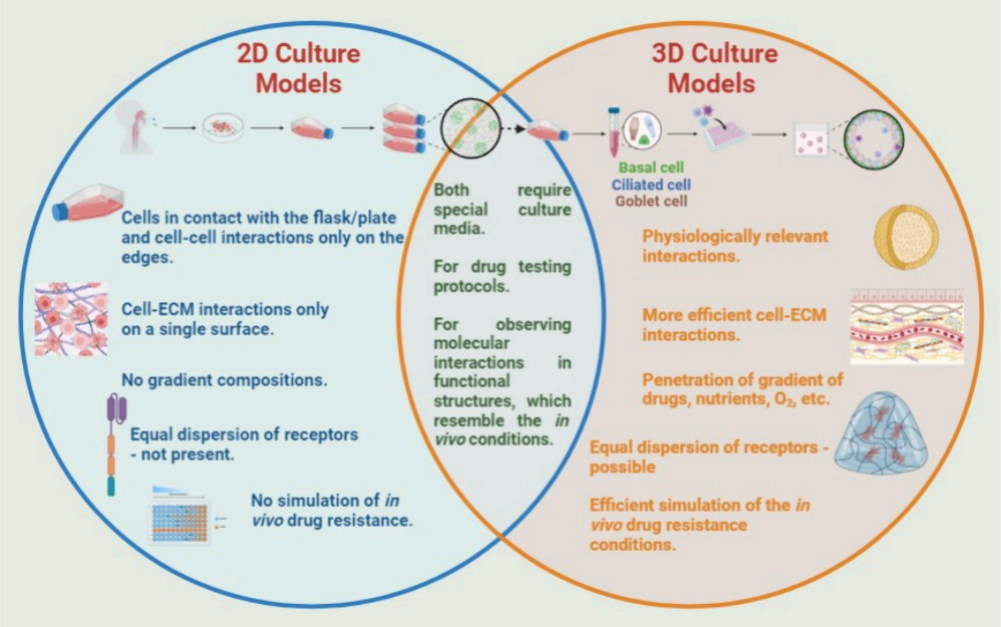
A diagrammatic comparison of 2D and 3D cell culture models, highlighting the key processes of their design, along with a list of their primary differences. Process: Bronchoscopy is used to obtain human airway biopsies. Human basal epithelial cells (HBECs) can proliferate on 3T3 murine fibroblasts by chopping biopsies into small explants. To grow in number, HBECs are further expanded on fibroblasts in a two-dimensional culture method. Next, fibroblasts and HBECs are sorted via differential trypsinization. After 1 week of culture, immune cells are added (if necessary) after they are sown in Matrigel. Twenty days after culture, organoids are generated (3D culture system).

The particular study objectives determine which model is best, highlighting the necessity of a customized strategy to deal with the complexity of biological systems. Integrating these models will probably help with more thorough and translational biomedical research as technology develops. A comparative representation of 2D versus 3D cell cultures is depicted in Figure 2.

## Microfluidic Organ-on-a-Chip

In the 1900s , two-dimensional cell culture technologies were developed that allowed the brief observation of the type of cell and the mechanism involved in a particular disease.^98,99^ The late 20th century saw an evolution in technological aspects such as 3D cell culture technologies, due to which it became more convenient to identify the precise cause of the disease.^100−102^ Along with the advancements in technology, the term microfluidics emerged, which meant the study of fluids on a microliter scale confined in micrometers, allowing an even more precise identification of a disease’s pathophysiology.^103^ The initial microfluidics devices have applications in host defense as well as enhanced understanding of microanalysis and molecular analysis. Microfluidic organs-on-a-chip are artificial systems containing a tissue that is allowed to mature inside microfluidic chips. The purpose of these chips is to directly and precisely mimic normal human physiological conditions and preserve tissue-specific functionalities.^104,105^ This is novel technology that has been created as a result of the advancements occurring in the field of engineering, with the main intention of assessing the pathophysiology of diseases in humans and creating novel therapeutic approaches to combat them. There are several different types of organs-on-chips, which is why it is difficult for researchers to standardize any one to treat all diseases. Hence, further knowledge about this is required.^106^ They have certain benefits, which include being easy to use and more convenient, having a miniaturized design, being highly sensitive, and having a high throughput design. The rapid development of microfluidic devices for organs-on-a-chip has been facilitated by 3D bioprinting.^107,108^ These technologies will provide future gateways for the enhanced discovery of patient-centric therapy and for regulating the disease microenvironment.^109,110^ Various organ-on-a-chip models have been developed until now, such as lung-on-a-chip, kidney-on-a-chip, brain-on-a-chip, heart-on-a-chip, skin-on-a-chip, liver-on-a-chip, etc., which makes it easy to identify and gain knowledge on the development of a wide range of diseases.^110^ A huge breakthrough has been created with the integration of sensors in these chips for real-time analysis in several experiments and biological procedures.^88^ There is a huge potential for organ-on-a-chip in the market, as there is no technology like it that has been developed until date. The market capital for organ-on-a-chip has grown to be approximately 41 million USD and it is estimated that up to 2026 it will shoot up to 303.6 million USD.^110,111^ The organ-on-a-chip ideation is still in the review stage, and only a few developments have occurred; to date, no regulatory body has given a classification to an organ-on-a-chip as a therapeutic agent, but it is expected that with more investigations it will soon gain attention in the scientific community.^112^

## Design of an Organ-on-a-Chip Model

Two-dimensional cell culture techniques that existed in the past had a variety of limitations, which include high cost, time consumption, failure rates, etc., as a result of which the advent of novel techniques with the idea of 3D cell culture environments came into play. An example of these techniques is microfluidic organs-on-chips, which can be applicable to a wide variety of areas such as tissues, engineering, drug discovery, etc.^113^ The regulation of the external and the internal environments of the cell culture systems is necessary, and the organ on chip technology can be used in combination with micromachining and cell biology to accurately simulate normal human physiological conditions.^114,115^ Several parameters such as dynamic mechanical stress, fluid shear, concentration gradients, and cell patterning must be taken into consideration while designing the organ-on-a-chip.^105,116^ Organ-on-a-chip can be further classified into single organ-on-a-chip or multiple organs-on-a-chip based on their applicability. Single organ-on-a-chip has a comparatively simpler design in contrast to multiple organs-on-a-chip. Both fall into the category of microphysiological systems, as they are designed to simulate human physiological conditions and provide insights into the pathophysiology of any disease.^104,117^ The decision whether to use single or multiple organ-on-a-chip depends on the functional requirements of the physiological processes. The degree of complexity should be kept as low as possible to the minimum required amount to avoid the additional factors that are undesired, which hamper the procedure and can affect the result.^104,118^ Another approach is to decide which functional tissue to incorporate within the organ on chip, which may include various types such as an engineered tissue, performed organoid, stem cells, etc. After being incorporated into the organ-on-a-chip, the cells are subsequently cultured into the microfluidic system in which they are allowed to mature, as a result of which the cells get matured into tissues. This accomplishes two tasks at once, i.e., it arranges and maintains cells in the organ-on-a-chip in the culture and it also allows the organ fluid to connect tissue components in a way that replicates their connectivity.^104,119^ There is no predetermined geometry for the organ-on-chip models; however, they can be classified into single-channel, double-channel, and multichannel chips based on their number of channels.^113,120^ The most frequently employed type of chips is double-channel chips, which comprise a centimeter-sized chip that contains two separate channels joined with each other by a porous membrane.^121^ The organ-on-a-chip structure can also have its respective subtypes that rely on the organ that it creates. These are namely solid tissue organ chips and barrier tissue chips. In solid tissue organ chips, the cells are allowed to mature as 3D tissue masses and can associate and interact with one another along with the cell culture medium. Examples include micropillars and microwells that are commonly used in tissues such as the liver.^122,123^ In barrier tissue chips, the device is designed in such a way that it forms a natural barrier between fluid compartments, allowing it to create selective transport processes across the barrier that is to be investigated. The examples of organs where these can be employed include the gut, lung, and the skin.^104,110^ For instance, a highly developed “lung-on-a-chip” device that accurately mimics the alveolar–capillary contact has been created. Human capillary endothelial cells are placed on the lower side of this novel design, which involves the culture of human alveolar epithelial cells over a flexible porous membrane coated with extracellular matrix (ECM).^124^ By introducing air through the upper channel and creating an air–liquid interface with the alveolar epithelium, breathing dynamics can be simulated.^125,126^ The vascular channel is used to circulate the culture medium, either with or without human immune cells, concurrently. The full-height side chambers are subjected to cyclic suction, which causes rhythmic relaxations and distortions in the porous membrane that is linked to the flexible polydimethylsiloxane (PDMS) side walls, simulating respiratory motions.^127^ The ensuing three-dimensional fluorescence confocal reconstruction demonstrates the complexity of the contact between the alveolar epithelium and the endothelium at the tissue–tissue level. The various types of materials used in the generation of organ on chips include hydrogels (gelatin, polyvinyl chloride, and polyethylene glycol), silicon (silicon nitride and silicon dioxide), metals (gold and titanium), and membranes (polycarbonate and polyethylene terephthalate).^113,128,129^

The concept of an organ-on-a-chip has attracted a lot of research attention lately, which is indicative of a concerted effort to transform in vitro systems for biological study. Research in this field uses a wide range of organs that are simulated on microfluidic systems with the goal of simulating the complex physiological dynamics of human organs in a lab setting. The goal of this field’s groundbreaking research has been to produce microscale devices that replicate organ-specific capabilities by integrating living cells. To obtain the best possible replication of in vivo circumstances, scientists have carefully examined a number of design factors, including cell culture methods, microchannel layout, and biomaterial choices. The information gathered from these initiatives highlights the potential uses of organ-on-a-chip models, including disease modeling, drug testing, and personalized medicine, in addition to outlining the technical details of these models. Organ-on-a-chip technology has been established as a transformative instrument in the field of biomedical research thanks to the rigorous experiments, statistical analysis, and peer-reviewed validations that uphold the scientific integrity of these investigations.^130^ The potential to advance therapeutic approaches and improve our comprehension of intricate biological processes seems to be growing as scientists work to improve and broaden these models. An investigation was conducted into the use of organ-on-a-chip technology in toxicology and drug screening. To evaluate drug metabolism and hepatotoxicity, the researchers employed a liver-on-a-chip model, showcasing the platform’s promise for successful and economical drug development.^131^ A further study examined the development and application of a microfluidic heart-on-a-chip model. The platform’s capacity to mimic cardiac tissue reactions was examined in the study, which offered insights into cardiovascular disorders and possible uses for drug testing.^132^ One study focused on neurodegenerative illnesses and created a brain-on-a-chip model to simulate the responses of neural tissue. The researchers showcase the platform’s usefulness in neuroscience research by demonstrating how it may be used to test therapeutic interventions and investigate the evolution of diseases.^133^

## Microfluidic Organ-on-a-Chip System: Design, Development, and Construction

### Primary Components

Microfluidics, living cell tissues, drug delivery stimulation, and sensing are the primary components involved in an organ-on-chip development.^134^ The microfluidic component involves a system of culture fluid input and waste liquid outflow during the culture process and refers to the use of microfluidics to deliver target cells to a predetermined area. Usually, miniaturization, integration, and automation characterize this component.^135^ The living tissue component is responsible for the spatial alignment of a specific cell. The 3D configured systems are created by the incorporation of biocompatible substances such as hydrogels, which resist mechanical harm and support the formation of 3D structures. The assembly of the extracellular matrix, the presetting and formation of vasculature, and the limitations of technology and cost mean that living cells in organ tissues are still primarily cultivated in 2D despite the fact that the 3D tissue structure more accurately simulates the in vivo situation compared to 2D models.^136^ To recreate the physiological milieu, which supports microtissue maturation and function, physical or chemical cues are needed for some tissues. Electrical stimulation, for instance, can promote cardiac tissue maturation.^137^ It is possible to derive several signal stimuli for drug screening methods.^138^ A transparent chip-based visual function evaluation system or an embedded sensing output component can be used as the sensing component for detecting and compiling the data. A cell system was created by Kane et al. to track cells in a 3D microfluidic environment. These tests used time-lapse imaging microscopy as a quality-control measure to evaluate cellular electrical activity.^139^ Multicellular OOACs were imaged by Peel et al. using automated techniques, resulting in comprehensive cell phenotypes and statistical models for measurements.^140^

### Microfluidics

Organ-on-a-chip (OOAC) technology, based on microfluidic devices created via microfabrication, has recently received a great deal of attention as a revolutionary in vitro organ model. Organ-on-a-chip technologies can be used to maintain cellular function and morphology and replicate organ interactions because microfluidic device technology makes it possible to mimic the physiological environment physically and chemically. The lung, liver, kidney, and gut are just a few of the organs and tissues whose functions have been mimicked in in vitro models thus far. Additionally, a body-on-a-chip approach has been proposed for the prediction of organ interactions, merging many organ functions on a microfluidic device. Precision handling and processing of microscale fluids is the focus of science and technology known as microfluidics.^141^ A “lab-on-a-chip” is a device that is frequently used to accurately manipulate microfluidic (10^–9^ to 10^–18^ L) fluids utilizing channels that range in size from tens to hundreds of micrometers. Its petite size, broad surface area, and high mass transfer make it ideal for microfluidic technology applications that require low reagent usage, predictable volumes, quick mixing rates, quick responses, and finely tuned control of physical and chemical properties.^142^ Microfluidics combines cell culture, sorting, and cell lysis with sample preparation, reactions, separation, and detection. These factors have increased interest in OOAC, and numerous fields such as chemical, biological, and material science are combined in OOAC technology. The OOAC is a biomimetic system with the capacity to control important parameters, such as concentration gradients, shear force, cell patterning, tissue boundaries, and tissue–-organ interactions.^143−145^ Simulating the physiological milieu of human organs is the primary objective of an OOAC.

### Techniques

Using semiconductor microfabrication techniques like photolithography and soft lithography, microfluidic devices can be employed for chemical reactions and analysis in microchannels and microstructures.^146^ Researchers have employed microfluidic devices in cell culture applications to bridge the significant gap between the in vivo and in vitro environments. Microfluidic techniques can be used to manage spatially and temporally liquid conditions, cell adhesion, and mechanical stimulation of cells. Recent years have seen a surge in interest in organ-on-a-chip technology, which replicates organ functions using this microfluidic technique. Tissue models and disease models for drug discovery employing organ-on-a-chip technology have been presented and are anticipated to serve as platforms for cell-based assays during drug development, particularly with the advancement of a differentiation induction approach for induced pluripotent stem cells (iPSCs). Early studies on organs-on-a-chip showed enhancements in functional activity through the perfusion culturing of 3D hepatocyte aggregations and the monitoring of reactions to shear stress by exposing vascular endothelial cells to medium flow in a microchannel.^147,148^

### Design Requirements

Fluid shear, concentration gradients, and dynamic mechanical stress are required on the chip, along with cell patterning.

#### Fluid Stress

An organ-on-a-chip, when combined with micromachining and cell biology, enables an accurate simulation of the in vivo system. Through micropump perfusion, microfluidics enables the dynamic culture of cells, facilitating the administration of nutrients and prompt waste discharge. Cells are situated in a dynamic environment that is more like in vivo settings than a static culture. Additionally, organ polarity is brought on by fluid shear stress.^149^ Importantly, an OOAC activates cell surface molecules and related signaling cascades to apply the appropriate physical pressure on endothelial cells’ typical biological processes.^150^ Similar to this, the addition of fluid to the OOAC device enables biological evaluations at the level of a single organ.^151^ The OOAC system summarizes movement using a straightforward “rocker” on a chip fluid motion, or, in organization-specific designs, a more intricate programmable “pulsatile” format arranged in a single loop.^122^ The fluid generally behaves as a laminar flow, producing a steady gradient of biological molecules that is regulated in both space and time at the microscopic level.

#### Concentration Gradients

Angiogenesis, invasion, and migration are just a few examples of biological activities that use different biochemical signals driven by concentration gradients.^152−154^ By adjusting flow rate and channel shape with the use of microvalves and micropumps to produce stable, three-dimensional (3D) biochemical concentration gradients, microfluidics can simulate intricate physiological processes in the human body.

#### Cell Patterning

For the creation of intricately shaped in vitro physiological models, microfluidics governs cell patterning. Cell patterning on the chip is influenced by surface alterations, templates, and 3D printing.^136,155,156^ The creation of hydrogel scaffolds with intricate pathways made possible by the 3D printing technique enables multiscale cell patterning. One benefit of 3D printing is that it enables user-defined digital masks, which are essential for the in vitro reconstruction of the cellular milieu and offer versatility in cell patterns. Using precise topological manipulations, techniques for achieving quick heterotypic cell patterning on glass chips have been developed by Li and colleagues.^157^

### Methods for Developing Lung-on-a-Chip Models

The three techniques most commonly used for fabricating lung-on-a-chip models include lithography-based microfabrication, the thermoplastic model, and 3D bioprinting.

#### Lithography-Based Microfabrication

A lung-on-a-chip model by Huh and co-workers and Stucki et al. used lithography-based microfabrication and is described below in detail. Sellgren and colleagues have also applied this method and created a model comprising an airway of epithelial cells. Lung fibroblasts and microvascular endothelial cells were cultivated on an air–liquid interface, in three vertically stacked compartments, each separated by a nanoporous membrane, on an air–liquid interface.^158^

#### Thermoplastic Model

A thermoplastic lung model has been designed and constructed by Humayun and co-workers using micromilling and solvent-bonding techniques. The lung airway milieu, interactions between smooth muscle and epithelial cells, and the supporting extracellular matrix (ECM) were all replicated by the chip. Epithelial cells were cultivated in the air–liquid interface, in a suspended hydrogel layer in place of a membrane, and in media reservoirs, respectively, in the upper, middle, and bottom chambers of the chip. The model was also dismantled to recover the suspended hydrogel for additional examination, which would be of assistance in researching how the smooth muscle and epithelial cells, and cellular matrix interact to form chronic lung diseases (CLDs).^114^

#### 3D Cell Bioprinting

**Figure 3:**
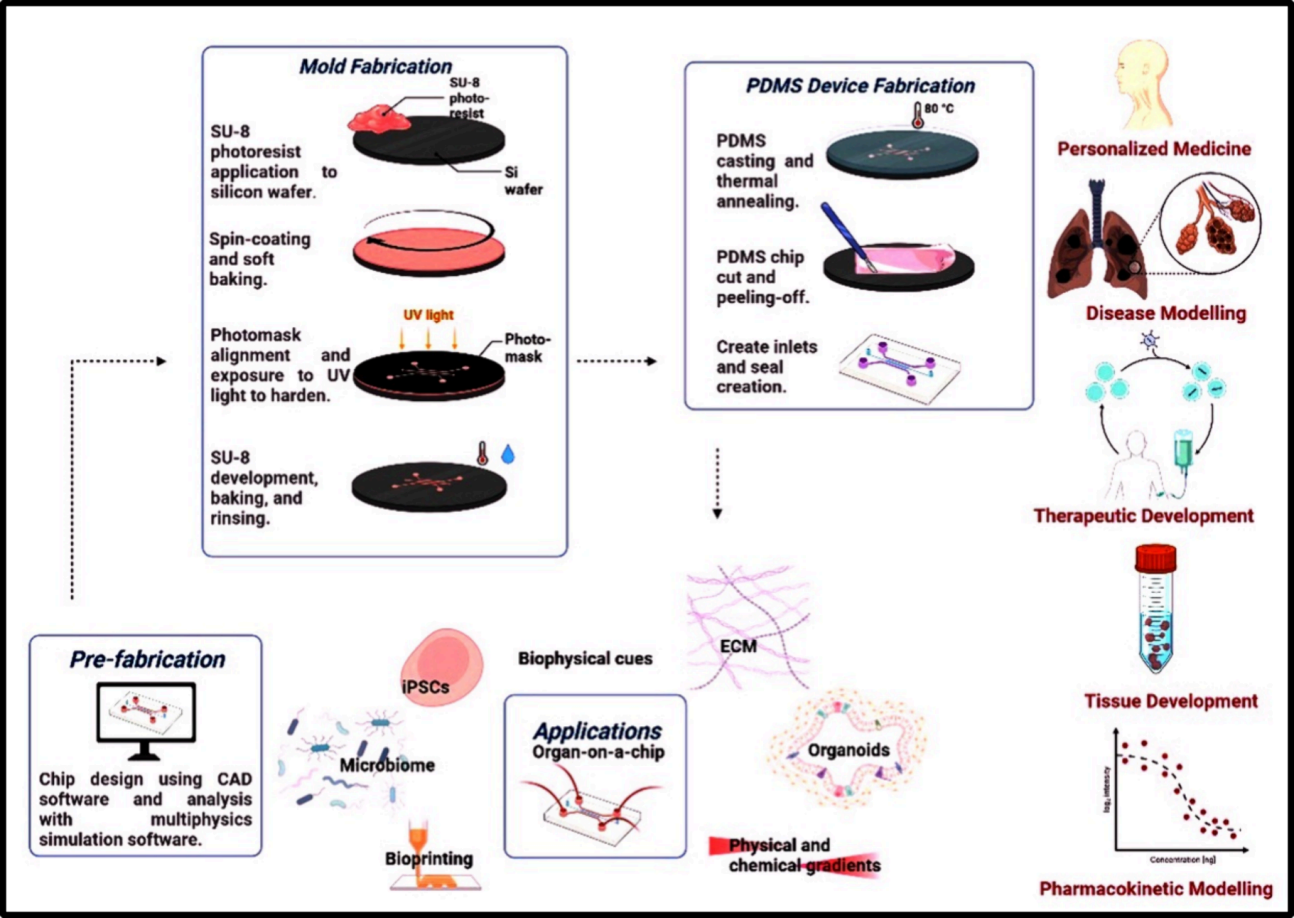
A schematic representation of PDMS-microfluidic organ-on-a-chip device fabrication along with the common applications of these models.

Park et al. created an airway-on-a-chip model with a vascular network utilizing 3D cell bioprinting. They used polycaprolactone, lung fibroblast bioink, endothelial cell bioink, and PDMS to create a vascular platform that had two side reservoirs, one for the epithelial cell (EC) bioink and one for the lung fibroblast (LF) bioink reservoirs, and was immediately 3D printed utilizing a cell-filled decellularized extracellular matrix (dECM) bioink. Microchannels for media flow and a location for PDMS bonding to the upper PDMS chip were used to separate the bioinks. A fully distinct model of the airway was created, producing a useful interface with the vascular network.^159^Figure 3 demonstrates a representation of the steps involved in the fabrication of a PDMS microfluidic organ-on-a-chip model along with the common applications of these models.

## Lung-on-a-Chip Models

*In vitro* simulation of the alveolar gaseous exchange is a difficult task; however, through scrupulous manipulation of fluid flow and gaseous exchange, microfluidics can establish extracorporeal pulmonary models. The blood–blood barrier (BBB), control of airway mechanical pressure, and the impact of shear force on pathophysiological processes have all been the subject of recent research.^160^ The first “lung-on-a-chip”, sometimes referred to as the “breathing lung”, was created by the Ingber research team at Harvard University.^60^ This device has a microporous membrane constructed of stretchy silicone, poly(dimethylsiloxane) (PDMS), which divides the two layers of the channel structure vertically. This is shown in Figure 3. The Ingber team created a microfluidic system that replicated the anatomy of the lung by growing alveolar epithelial cells on the upper surface and vascular endothelial cells on the bottom surface. Through alteration of the internal pressure of the channel on both sides of the main channel at a given cycle, the physiological expansion and contraction motions were simulated. Using this apparatus, the authors mimicked inflammatory responses in which vascular endothelial cells strongly express the integrin ligand (ICAM-1) following cell exposure to bacteria and tumor necrosis factor (TNF). In addition, neutrophils moving through the vascular side channel became linked to the vascular endothelial cells after ICAM-1 was expressed. They then moved through the vascular endothelial cells and the membrane pores to the alveolar epithelial cell surface side and phagocytosed the bacteria. Addition of interleukin-2 (IL-2) resulted in the disease model of pulmonary edema.^161^ The amount of nanoparticle uptake into the blood vessel side of the device was increased by the stretching movements of the membrane according to a toxicity test utilizing nanoparticles. Similar outcomes were attained in an animal experiment carried out under comparable circumstances. Ingber et al. also developed a disease model that used the apparatus to replicate the signs and symptoms of pulmonary edema for a different investigation.^161^ Inhibition of extravasation was seen when a low-molecular-weight medication was used to treat pulmonary edema in this illness model, which was comparable to what was seen in an animal model of pulmonary edema. As a result, applications as an in vitro disease model have also been proposed. Other organs including the kidney and intestine have also made extensive use of this device.^162−164^ A lung chip that resembled the lung parenchyma was described by Stucki et al. The system was the first elastic membrane expansion model to replicate breathing, and it comprised an alveolar barrier and 3D cyclic strain to simulate respiration.^165^ To determine their applicability as a physiological model, Humayun et al. grew airway epithelium and smooth muscle cells on various sides of a hydrogel membrane. The system was used as a physiological model of chronic lung disease along with microenvironmental cues and toxin exposure.^114^

The development of novel medications has been hampered by the absence of trustworthy and functional models to simulate respiratory disorders. In a study to evaluate the effectiveness of lung-on-a-chip and address this gap, Huang et al. developed a three-dimensional porous hydrogel made of gelatin methacryloyl (GelMA), which was attached to a segmented chip device. The model included a 3D configuration, with the epithelial layer being formed by primary human alveolar epithelial cells (hAECs) packed on the sac surfaces. The utilization of the GelMA structure allowed for an accurate simulation of the human lung environment due to its strong resemblance to the natural alveolar sacs, which were composed of human alveolar sacs and contained sac-like pores. Using pictures from scanning electron microscopy, the morphologies of the hAECs were investigated on days 3, 7, and 14. In comparison to the planar construction, the 3D culture was observed to provide the most effective geometry for cell spreading and proliferation, and both structures exhibited a strong resemblance to normal human lung tissue.^166^ In order to verify the validity of this model, the effects of smoking more than ten cigarettes were observed and the cells were further cultured for 24 h, after which dead cells in the alveoli started to increase in number and the lifespan of hAECs decreased in comparison to the control groups.^167^ A poly(lactic-*co*-glycolic acid) (PLGA) electrospinning nanofiber membrane was created by Yang et al. as a chip matrix for cell scaffolds. This simple system makes lung tumor precision therapy and tissue engineering approaches applicable.^168^ By exchanging fluid and media, a 3D airway culture model by Blume and co-workers replicated pulmonary interstitial flow. This made it possible to do more thorough physiological research on the epithelial barrier. This model combined many chambers for better integration and used a stent with a permeable filter as a single tissue culture chamber. In the lung-on-a-chip system, pressure can be applied to the alveoli and associated capillaries, giving a shear flow profile while emulating lung gas–liquid interfaces and respiratory dilation through the microfluidic system. This models the lung environment in a realistic manner.^169^ These lungs-on-a-chip are also utilized for their applicability as implantable respiratory assistance devices. Xu et al. investigated various chemotherapeutic medicines while simulating the microenvironment of lung cancer using cancer cell lines and primary cancer cells on a microfluidic chip platform.^170^ Another recent study used a “small airway-on-a-chip” device to simulate asthma.^171^ Lung assistance devices (LADs) were created by Peng et al. to enable more gas exchange in the placenta for premature newborns experiencing respiratory insufficiency. In the umbilical arteries and veins, the idea of large-diameter channels was realized, giving the LAD a high extracorporeal blood flow. Because clinical trials for determining umbilical vasodilation thresholds were unethical, this provides additional value. This study was the first to objectively measure the harm caused by catheter extension to the umbilical vessels.^172^ To enhance gas exchange, Dabaghi et al. microfabricated double-sided gas delivery systems for microfluidic blood oxygenators. Comparing double-sided devices to single-sided ones, oxygen consumption increased to 343%.^173^

## Recent Reports on Pulmonary Organ-on-a-Chip Models

Pulmonary organ-on-chip models have garnered significant attention in recent times and have been developed for several applications, from drug development and drug testing to the study of pulmonary diseases and infections. Multiple such models have been designed to date, and Table 1 provides insights into the reported pulmonary organ-on-chip models, utilized for the investigation of many disease processes. The use of lung-on-a-chip models has indicated a great deal of promise for the design and discovery of effective therapeutic agents for various pulmonary conditions. The study by Huh and co-workers also investigated the role of angiopoetin-1 in preventing IL-2-induced vascular leakage. Angiopoetin-1 also inhibited the formation of paracellular gaps, despite a rhythmic mechanical strain, which activates the transient receptor potential vanilloid 4 (TRPV4) ion channels.^161^ Consequently, it causes an increase in the permeability of the alveolar–capillary interface. This eventually results in vascular leakage in the lungs.^174^ In the presence of cyclic mechanical strain, a TRPV4 channel blocker, GSK2193874, was administered into the microvascular channel to limit the IL-2-induced increase in the vascular permeability. These results imply that patients with pulmonary edema may benefit from effective treatment choices.^175^ A bromodomain containing protein 4 (BRD4) inhibitor was evaluated on an inflamed human small airway chip, and it was shown that neutrophil adhesion was suppressed as a result of lower expression levels of adhesion molecules (E-selectin, VCAM-1, and ICAM-1). Cytokine genes like IL-8, MCP-1, GROa, and IL-6 were also significantly downregulated by this agent, and there was a marked reduction in neutrophil chemokine and granulocyte-macrophage colony stimulating factor (GM-CSF).^176^ Navarixin (MK-7123), a CXC chemokine receptor-2 (CXCR2) antagonist inhibited the human rhinovirus (HRV)-induced asthmatic responses.^177^ For individualized lung cancer treatment, Xu and colleagues created a microfluidic chip-based 3D coculture model for assessing drug sensitivity. The tools assessed the sensitivity of cell lines and primary cells to various anticancer medicines. To demonstrate the potential for individualized therapy, accurate screening of dose-related, single, and combined medication regimens was performed. The study showed that the approach, when used for drug sensitivity testing, was straightforward, extremely sensitive, high-throughput, and time-efficient. The model has been shown to be useful for a variety of lung cancer cell lines as well as primary cancer cells, assisting chemotherapeutic professionals in determining the best chemotherapy regimen for treating lung cancer.^170^ A microfluidic lung-on-a-chip model of asthma and COPD, developed by Jain et al., assessed the in vitro human thrombotic responses to a novel pharmacological agent, parmodulin-2, which is a protease-activated receptor-1 inhibitor.^171,178^ When evaluated on the asthmatic on-chip model, tofacitinib, a Janus activated kinase (JAK) inhibitor used to treat rheumatoid arthritis, restored the asthmatic alterations caused by IL-3.^179^ This was accomplished by reducing the release of the cytokines, granulocyte colony-stimulating factor (G-CSF), and granulocyte-macrophage-colony-stimulating factor (GM-CSF); suppressing goblet cell hyperplasia; and restoring the regular cilia beating frequency. The clinical observation that dexamethasone inhalation treatment fails to improve the symptoms of moderate to severe asthmatic patients was further reinforced by the discovery that treatment with dexamethasone is ineffective in IL-13 exposed chips. These findings support the possible application of JAK inhibitors for allergic rhinitis.^180^

**Table 1:** Detailed Representation of the Various Pulmonary Organ-on-Chip Models Reported, Techniques Used in Their Design and Development, and Primary Findings^a^

research group	technique used	main findings
lung physiology		
Huh et al.^60^	Introduction of air in the epithelial chamber, microfluidic system that replicated the anatomy of the lung by growing alveolar epithelial cells on the upper surface and vascular endothelial cells on the bottom surface, respectively. By altering the internal pressure of the channel on both sides of the main channel at a given cycle, the physiological expansion and contraction motions were simulated.	Air in the epithelial chamber allowed the cells to survive longer, increased pulmonary surfactant generation, increased electrical resistance over the various tissue layers, improved structural integrity, and normal barrier permeability.
	Injected fluid containing bloodborne immune cells into the vascular channel and a strong pro-inflammatory mediator, tumor necrosis factor-a (TNF-a), into the upper alveolar channel. Leukocyte adhesion molecules and intercellular adhesion molecule-1 (ICAM-1) were measured.	Activation of endothelial cells on the lower channel in the presence of physiological mechanical strain. The activated endothelium in the vascular microchannel made it easier for neutrophils to stick together. Following that, neutrophils crossed the capillary-alveolar barrier.
Sellgren et al.^181^	In the side chambers, vacuum was supplied to produce consistent physiological cyclic strain (10% at 0.2 Hz).	Well-differentiated primary human tracheobronchial epithelial cells duplicated physiological processes at an air–liquid interface. The endothelial cells in the lower chamber used cell alignment to mimic the natural response seen in blood arteries and cultivated endothelium in vivo.
Stucki et al.^182^	Cyclic mechanical stretching.	Cyclic mechanical stretching had a considerable impact on the permeability of the epithelial barrier. Dynamic mode culture significantly increased the metabolic activity of the cultivated alveolar cells and generated more of the inflammatory marker IL-8.
Zamprogno et al.^183^	An array of stretchable alveoli using a stretchable biomembrane made up of collagen and elastin to simulate physiological activities.	The membrane replicated the composition, shape, transport, and mechanical characteristics of the alveolar barrier and allowed cells to be cultivated at the air–liquid interface. The structural stability and elasticity, provided by the collagen-I and elastin, were crucial for recreating and enduring the continual physiological breathing motions.
toxicological studies		
Huh et al.^59,60^	Exposure of the alveolar epithelium to silica nanoparticles resulted in enhanced expression of ICAM-1 and activation of the endothelium beneath it. The nanoparticles were then absorbed into the microvascular channel. The intracellular production of reactive oxygen species (ROS) was estimated in order to study the cellular oxidative stress response induced by the nanoparticles. By moving nanoparticles through the membrane from the alveolar to the vascular channel, the lung-on-a-chip mimicked the movement of particles over the alveolar–capillary interface.	The significance of mechanical movements in the functionality of the lungs is demonstrated by the fact that exposure to nanoparticles and natural breathing motions both produced and accelerated harmful effects on the lungs.
Zhang et al.^184^	Toxicity testing model with three parallel channels, including human endothelial and alveolar cells on the sides with a centered matrigel layer. Titanium and zinc oxide nanoparticles were employed to assess their effects on cellular morphology, ROS generation, epithelial and endothelial cell apoptosis, and junctional protein expression.	A resourceful model for safety assessment of nanoparticles, food, environmental particles and drugs.
asthma and chronic obstructive pulmonary disease		
Benam et al.^171,185,186^	Small airway chip supporting a differentiated mucociliary and bronchiolar epithelium with an underlying lung microvascular endothelium, with immune cells motioning through the underlying fluid flow.	IL-13 treatment of the small airway resulted in an increase in the number of Goblet cells and inflammatory cytokine release. Moreover, the cilia beating frequency decreased. These findings are normally seen in asthmatics.
	Exposure of airway epithelium to polyinosinic-polycytidylic acid.	Pro-inflammatory response, as commonly observed in asthma exacerbation.
	Healthy and COPD epithelial airway cells stimulated with bacterial infection mimic, lipopolysaccharide (LPS) endotoxin, or viral mimic.	Cytokine M-CSF and IL-8 were seen at higher levels in COPD chips compared to the healthy cells. The fact that M-CSF was exclusively up-regulated in the presence of the viral mimic PolyI:C suggested that it may serve as a novel biomarker for acute viral exacerbation in COPD patients.
Nesmith et al.^187^	Bronchial smooth muscle cells on elastomeric thin films for a human airway musculature-on-a-chip.	Responses of an asthmatic musculature to IL-13 were successfully reproduced. The model additionally analyzed a Rho pathway inhibitor, HA1077, which reduced the asthmatic response.
Villenave et al.^188^	A fully differentiated mucociliary bronchiolar airway epithelium maintained by a microvascular endothelium on a human airway chip.	Pro-inflammatory response seen upon infecting the airway chip with human rhinovirus (HRV). The response was characterized by time-dependent cytokine release and extravasation of circulating neutrophils.
	IL-13 infection to the HRV-infected airway chip.	Adhesion molecule upregulation in endothelial cells and enhanced neutrophil recruitment.
pulmonary thrombosis		
Jain et al.^60,161,178^	A modification of the existing lung-on-a-chip; the lower vascular channel was lined with vascular endothelial cells. A vascular lumen with human whole blood perfusion was created.	Rapid platelet recruitment and thrombus formation due to TNF-α stimulation, similar to the inflamed microvessels, under in vivo conditions. Tear-drop shaped thrombi and rapid platelet binding dynamics were also noted.
pulmonary edema		
Huh et al.^161^	A microdevice to assess the toxicity of IL-2. When IL-2 was infused through the vascular channel, there was a constant leakage of clear fluid into the alveolar compartment.	Mechanical breathing motions play an eminent role in IL-2-induced vascular leakage, eventually causing pulmonary edema. IL-2 perfusion into microvascular channel also causes fibrin clot formation, simulating an in vivo clot.
idiopathic pulmonary fibrosis and/or alveolar microinjuries		
Felder et al.^189^	Microfluidic lung chip with an epithelial wounding structure used to investigate the effect of gastric contents on the alveolar epithelium. Alveolar microinjuries were also reproduced in an effective way.	Gastric content exposure resulted in a disruption of barrier integrity of the alveolar epithelium. This led to pulmonary fibrosis.
Felder et al.^190^	Development of a breathing lung-on-a-chip and a wound-healing assay to evaluate the effects of human hepatic growth factors (rhHGF).	Cyclic mechanical stretch greatly slowed the healing of wounds; however, this was partially reversed once rhHGF was administered.
lung cancer		
Hassell et al.^191^	Human nonsmall cell lung cancer model to study cancer dynamics and tumor microenvironment, along with evaluating the response of tyrosine kinase inhibitors.	The growth of tumor cells was markedly inhibited by the presence of a cyclic mechanical strain that mimicked breathing patterns. When there was no motion, the tumor cells, which were contained in a small area, grew above and below the alveolar epithelial layer to replace it, suggesting the presence of a positive feedback loop that promotes the development of tumor cells. The study also observed cancer cell invasion and migration into the vascular channel, both of which were significantly inhibited by breathing motions.
Yang et al.^168^	Lung-on-a-chip model with PLGA cocultured with human NSCLC A549 cell lines. Gefitinib was evaluated, and insulin-like growth factor (IGF-1), secreted by HFL1 cells, was also investigated.	The study reported a reduced sensitivity of the tumor cells to the drug, and it was demonstrated that A549 has the potential to promote tumor cell invasion by promoting apoptosis or endothelial cell death.

a These research reports have been segregated into several sections, according to their applications.

## Modeling of SARS-CoV Infections on Chip

Infectious diseases continue to be a significant concern for public health on a global scale. With the ongoing COVID-19 pandemic caused by the infection of severe acute respiratory syndrome coronavirus 2 (SARS-CoV-2), there has been a growing focus on dedicating resources to investigating treatments that target the spike glycoprotein of the virus and developing different types of vaccines. Organ-on-a-chip devices present a groundbreaking approach to simulate viral infections in human tissues, enabling efficient screening of antiviral treatments through high-throughput methods. The alveolar–capillary barrier has the prime responsibility of maintaining gaseous exchange, as mentioned earlier, along with preventing the spread of viruses. SARS-CoV-2 infection involves the lung as the primary organ, and the condition ranges from mild lung injury to multiorgan failure.^192,193^ The progression of severe COVID-19 often leads to a gradual failure of the respiratory system, which contributes to fatalities. This failure is caused in part by widespread damage to the tiny air sacs in the lungs (known as alveoli), as well as inflammation and the development of pneumonia.^192^ Consequently, the utilization of lung-on-a-chip 3D models can effectively simulate lung injuries and immune responses triggered by the SARS-CoV-2 virus. These models accurately replicate the flow of fluids in both healthy and diseased conditions.^194^ Zhang and colleagues successfully developed a chip-based model that replicated human alveolar conditions and allowed for the study of SARS-CoV-2 infection and immune responses, providing insights into the preferential infection of the virus of epithelial cells and the inflammatory reactions triggered by the presence of immune cells. They developed a chip-based model called human alveolus-on-a-chip, which allowed them to infect the model with SARS-CoV-2. The chip was created using conventional soft lithography techniques and consisted of two chambers: an upper chamber representing the alveolar space lined with human alveolar epithelial type II cells (HPAEpiC) and a lower chamber representing the lung microvasculature lined with lung microvascular cells (HULEC-5a), which were separated by a thin PDMS membrane. When SARS-CoV-2 was introduced into the alveolar chamber, spike protein expression was observed in the epithelial cells but not significantly in the endothelialized chamber. This suggests that the virus has a higher affinity for infecting cells with a higher expression of ACE2 receptors, such as epithelial cells. Furthermore, immune cells were introduced into the chip by infusing human peripheral blood mononuclear cells (PBMCs) into the lower vascular chamber. The presence of PBMCs led to elevated expression of cytokines including interleukin-1β (IL-1β), IL-6, IL-8, and tumor necrosis factor-α (TNF-α) following SARS-CoV-2 infection. This confirmed the recruitment of PBMCs and an enhanced inflammatory response in lung tissue, similar to what occurs in the human body during infection. To analyze the response of the cells to SARS-CoV-2 infection, RNA sequencing was performed on the HPAEpiC and HULEC-5a cells. Sequencing confirmed higher levels of viral replication in HPAEpiC cells compared to those in HULEC-5a cells, which was consistent with the findings from Western blot and immunostaining analysis. The findings indicate that the human alveolar epithelial cells are more susceptible to SARS-CoV-2 infection than microvascular endothelial cells.^194^ Si and co-workers constructed a human lung airway-on-a-chip model to facilitate the study of SARS-CoV-2 infection. The chip consisted of a porous membrane coated with extracellular matrix (ECM), along with an airway channel and a vascular channel. The device successfully supported the differentiation of lung airway basal stem cells into various specialized cell types found in the airway, including mucociliary cells, ciliated cells, goblet cells that produce mucus, club cells, basal cells, and pseudostratified epithelium. They reported that the highly differentiated lung epithelial cells in the airway chips exhibited elevated expression levels of the angiotensin-converting enzyme (ACE2) and transmembrane protease serine-2 (TMPRSS2). These proteins play a crucial role in facilitating cellular entry and infection by influenza viruses. The findings suggested that the human lung airway chips could be utilized to investigate the effectiveness of approved drugs for the treatment COVID-19 infection.^195^ Some of the recent studies related to the use of organ-on-a-chip models for SARS-CoV-2 are summarized in Table 2.

**Table 2:** A Summary of Some of the Recent Research Reports Encompassing the Role of Organ-on-a-Chip Models in SARS-CoV-2-Related Investigations

research group	technique used	main findings
Zhang et al.^196^	They intended to develop a model that could simulate SARS-CoV-2 infection and to study the human responses in vitro using a microengineered lung chip device.	They observed that viral infections could trigger antiviral or immune responses in the host cells and performed gene ontology (GO) analysis to identify the particular genes at risk, after which they found that IL-16, IL-11, and CXC motif chemokine ligand 11 (CXCL11) were at higher risk.
Domizio et al.^197^	It has been hypothesized that macrophages and endothelial cells are involved in the type I interferon (IFN) responses involved in SARS-CoV-2, which is why they developed a lung-on-chip model that could simulate the alveolar-capillary interface and endothelial cell involvement in SARS-CoV-2 to study this.	Analysis showed that endothelial cells and macrophages with infection with SARS-CoV-2 activates cyclic GMP-AMP synthase-stimulator of interferon genes (CGAS-STING) signaling by mitochondrial DNA leading to cell death and subsequent type I IFN release.
Si et al.^198^	They built a human lung-on-chip, which was made of an extracellular matrix (ECM)-coated porous membrane, airway channel, and vascular channel to study the SARS-CoV-2 infection.	The device was shown to accurately differentiate the lung airway system into goblet cells, basal stem cells, mucociliary ciliated cells, etc., which allowed proper simulation of lung airway function. These portions were shown to express large amounts of angiotensin-converting enzyme (ACE2) and transmembrane protease serine-2 (TMPRSS2), which are important for cellular entry of the virus. Through this, it was proved that lung airways of humans can be employed to study viral infections.
Cao et al.^199^	They created a 3D alveolus-on-chip device comprised of three channels made of collagen gel channel in the center surrounded by two cell cultures. Polyinosinic:polycytidylic acid [Poly(I:C)], which is an IFN inducer and is commonly used as an immunostimulant to viral infections, was used to mimic SARS-CoV-2 infections.	This device could more accurately simulate the viral microenvironment and microarchitecture than the conventional 2D monolayer cell culture which allowed for better investigations into this.
Thacker et al.^200^	They recreated lung-on-chips in which CD14+ cells could be added to the human alveolar epithelial cells to study the immune system response. The device was composed of two channels separated by a PDMS membrane. The comparison was made of the differences between alveolar epithelial cells and vascular endothelial cells when SARS-CoV-2 infection was induced.	They observed that after infection with SARS-CoV-2, endothelial cells from infected lung-on-chips showed significant upregulation of TNF-α, IL6, and IFN-β and more modest increase ininterferon-λ 1 (IFNL1) and IFNL3 expression.
Guo et al.^201^	They developed a gut-on-chip that consisted of a human intestinal epithelial layer and a vascular endothelial layer separated by an ECM-coated PDMS membrane. In this, human colon adenocarcinoma (Caco-2) cells and human colorectal adenocarcinoma were cocultured in the upper channel, while human umbilical vein endothelial cells (HUVECs) and circulating immune cells were cultured in the lower channel under fluid flow. They investigated replication of SARS-CoV-2 in epithelial cells.	Through analysis, it was observed that the size of endothelial cells was decreased due to vascular endothelial cell injury following viral infection. This could partially explain the pathogenesis of COVID-19-associated coagulopathy or vascular thrombosis.

## Current Applications and Limitations of Lung-on-a-Chip Models

Microfluidic organs-on-chips are designed to mimic the functioning of lung tissues. These comprise microengineered systems with an integrated membrane outlined by a microfluidic setting. The microfluidic channels are able to cause the formation of fluidic characteristics of the tissue at the in vivo level, thereby improving the tissue functioning.^202,203^ Lung-on-a-chip technology makes it convenient to mimic breathing movement and restore the capacity of the lung tissues by employing a thin and stretchable PDMS membrane. A challenge involved in this is the air–blood barrier and its complex structure, which is quite difficult to mimic under in vitro conditions.^204^ Due to their ability to mimic normal breathing processes, lungs-on-chips have various applications. They are able to focus on studying the effects of air exposure on cell viability and the integrity of the cell layer and to investigate the mechanical stress of the epithelial cells of the lungs.^105,165,205^ Furthermore, the lungs-on-chips can be used to conduct inhalational assays to determine whether the individual has ingested tobacco or aerosol. Through this, acute liver injury (ALI) can be determined, which is a causative factor for asthma, chronic obstructive pulmonary disease (COPD), etc., as respiratory diseases are the main causes of mortality throughout the globe and their prevalence depends on the environment of the individual.^206,207^ Future prospects of this technology include its transformation into 3D technology with to increase the sensitivity and precision of the analysis. It can be employed for 3D ALI cell culture, investigations into disease pathophysiology, disease repair and regeneration models, toxicity testing, drug efficacy studies, etc.^208−210^ Another application of lung-on-a-chip includes the stimulation of normal physiological breathing by stretching the cells by introducing a vacuum that leads to the deformity of the elastic membrane to which the cells are attached.^211−213^ Creating and enhancing the organ-on-a-chip approach will take a lot of time and effort, and it is proposed that further studies into this will create a wide range of applications, which will lead to the creation of novel therapeutics to combat several diseases.

The lung parenchyma is composed of several small alveoli that are organized in a 3D structure.^214,215^ The deformity of the alveolar airspace and the septa occurs due to the continuous exposure of this environment of the lung to the breathing movement of smaller alveoli. Due to the complicated parenchymal architecture and the varying interstitial area width, mechanical stress results in the lungs.^216,217^ Thus, as a result the precise lung alveolar function is distinctive and challenging to replicate in vitro due to its dynamic environment.^204^ In addition to the several benefits of organs-on-chips, they possess a variety of disadvantages, including the dominance of the surface effect, which is considered a limitation of organ on chip because the dimensions of fluids are very small. Due to this, the product to be analyzed may get absorbed and lead to poor quality of analysis.^218−220^ As a result of this, the fluids do not mix effectively, as laminar flow is required where the fluids get converged. Another drawback includes the requirement of specialized equipment in certain studies to be able to provide accurate findings.^221^ The organ on chip technology is developing rapidly; however, its applicability in humans is still lacking. The most used material in the development of these chips is polydimethylsiloxane (PDMS), but its also possesses some drawbacks sicj as the thickening of its film as compared to normal in vivo morphology, which might lead to a reduced absorbance of small hydrophobic molecules and thus toxicity. Hence, it is necessary to recognize appropriate alternative materials.^116,222,223^ These limitations hinder the proper functioning of organs-on-chips and need to be addressed before designing an organ-on-a-chip to ensure its proper efficacy. Table 3 provides an overview of the applications, advantages, and limitations of lung-on-a-chip models.

**Table 3:** An Overview of the Applications, Advantages, And Limitations of Lung-on-a-Chip Models

aspect	applications	advantages	limitations
drug testing	evaluation of drug toxicity and efficacy	provides a realistic microenvironment for drug testing	limited representation of the entire systemic response
disease modeling	Modeling respiratory diseases like asthma, COPD, lung cancer, etc.	mimics disease-specific microenvironments	simplified representation compared to the in vivo conditions
respiratory physiology	studying lung-specific physiological responses	allows dynamic monitoring of cellular responses	may lack the complexity of the entire respiratory system
inhalation toxicology	assessing the impact of inhaled substances on lung cells	enables controlled exposure to airborne substances	difficulty in replicating the diverse cell types
personalized medicine	tailoring treatments based on individual patient responses	facilitates patient-specific drug testing	challenges in incorporating genetic and individual variabilities
microenvironment control	investigating the influence of microenvironment factors	offers precise control over biochemical and biomechanical cues	may struggle to replicate certain aspects of the complex in vivo microenvironment
air–liquid interface culture	studying the effects of air exposure on lung cells	allows simulation of the physiological conditions at the air–liquid interface	may require specialized equipment and expertise for setup and maintenance

## Challenges and Prospects of Pulmonary Organ-on-a-Chip Systems

Organ-on-a-chip has the advantage of understanding the complexity of the tissues of a specific organ, which provides the possibility of designing future therapies that have great future prospects and challenges at the same time.^105,224^ Restrictions on the use of organs-on-chips also occur in their process of formation, and these restrictions must also be addressed for this technology to become highly sensitive and efficient in the future. These include biological, technical processing errors, errors in cell culture of the chips, lack of specific machinery for their generation, etc.^224−226^ Other challenges include the appropriate scaling of the organ tissue sizes and counting their cell numbers using the device.^227,228^ The most recognized challenge of organ-on-a-chip in today’s world is its limited availability in the market, which will need to be addressed in the future. Several other limitations of organ-on-chips include the low-throughput characteristics of perfusion into the body, sampling problems, and fabrication and scale-up issues. As this technology relies completely on the availability of chips, which are an advanced technology, a deficiency can lead to decreased supply and increased demand.^224,229^ Along with this, the limited supply of sensors also poses a challenge in the overall supply of organs-on-chips. As these are devices, technical issues are likely to occur that need continuous monitoring and regular maintenance. Certain examples of these include drug absorption capacity toward PDMS, which is a material used in organ-on-a-chip generation; sterility maintenance; bubble formation; maintenance of biosensors; flow rate control between platforms; etc.^227,230^ Furthermore, their construction also poses a problem, as it involves the utilization of novel technologies such as bioprinting, high-resolution imaging, etc., and the availability of these techniques is quite important for their development. It is expected that soon enough with the advancement of technology it will be feasible to produce these devices without any construction-related hurdles.^231^ As these machines are created with the help of technology, their monitoring in terms of functioning is very crucial to ensure their appropriate effect ,as any slight deviation may lead to the possibilities of risk or harm to the individual.^232,233^ These devices may lead to difficulty in data reproducibility, and sometimes it may also be difficult to interpret the data to be analyzed. High-throughput data is generated due to organ-on-a-chip devices, and it is necessary to store these data and interpret them, which also is an obstacle toward organ-on-a-chip devices.^234,235^

The organ-on-a-chip devices are extremely accurate in predicting the relationship between the tissue of the organ and in vivo effects, which cannot be accurately performed by 2D or even 3D cell culture techniques. Therefore, these devices have been recognized as game-changers in the context of biomedical engineering as they bridge these fields together.^236,237^ It is also expected that and organ-on-a-chip may be designed in such a way that it may be able to perform pharmacokinetic and pharmacodynamic analyses for potential therapeutics.^236^ The process of adding amalgams onto the organs-on-chips is called amalgamation, and it has been hypothesized that this process can improve the sensitivity of the devices in the near future. These devices have created huge capital in the market, as there is a high requirement for these devices in the appropriate diagnosis and treatment of various diseases. It is also expected that the market demand for these devices will increase significantly in the future.^110^

## Conclusion

The organ-on-a-chip devices emerged in early 1990 with 2D devices under the terminology of miniaturized chemical analysis instruments and were used for analytical purposes. With time and the advent of technology, they have been repurposed for treating human diseases with high accuracy and precision and have wide potential in the future. These devices are able to facilitate the functioning of several organs, which allows the regeneration of organ function along with their normal physiological microenvironment and increases vascular perfusion into these organs in a way that 2D and 3D cell culture systems cannot achieve. Since their arrival on the market, they have provided promising therapeutics to cure a variety of diseases and began to grow in sophistication with the advancement of technology. Furthermore, they make it possible to analyze and provide imaging of in vitro biological cells, tissues, and organ function in high resolution, making it easy to identify the pathogenesis of several diseases and design therapeutics accordingly. However, they pose great challenges along with their high benefits in terms of processing, availability of materials, monitoring, etc. Sensors have been integrated into these devices in order to increase sensitivity and accuracy in several experimental analyses and biological investigations.

## Data Availability

The data can be made available by requesting the corresponding authors.

## Abbreviations

2D: two-dimensional
3D: three-dimensional
ACE2: angiotensin converting enzyme
ALP: advanced lipid peroxidation end products
ANS: autonomic nervous system
ASCs: adult stem cells
BBB: blood–blood barrier
BRD4: bromodomain containing protein-4
Ca^2+^: calcium ions
Caco-2: human colon adenocarcinoma cells
CD14+: cluster of differentiation-14
CGAS-STING: cyclic GMP-AMP synthase–stimulator of interferon genes
CLD: chronic lung diseases
COPD: chronic obstructive pulmonary disorder
COVID-19: coronavirus disease 2019
CXCL11: CXC motif chemokine ligand 11
CXCR-2: CXC chemokine receptor-2
dECM: decellularized extracellular matrix
DEP: diesel exhaust particles
DNA: deoxyribonucleic acid
EC: epithelial cell
ECM: extracellular matrix
GBD 2015: Global Burden of Disease 2015
G-CSF: granulocyte colony-stimulating factor
GM-CSF: granulocyte-macrophage colony-stimulating factor
GO: gene ontology
H1N1: influenza A virus subtype H1N1
HPAEpiC: human alveolar epithelial type II cells
HRV: human rhinoviruses
HUVECs: human umbilical vein endothelial cells
ICAM-1: intracellular cell adhesion molecule 1
IFN-ϒ: interferon-ϒ
IFNL1: interferon-λ 1
IGF-1: insulin-like growth factor
IL: interleukin
iPSCs: induced pluripotent stem cell
JAK: Janus activated kinase
LAD: lung assistance device
LF: lung fibroblast
LPS: lipopolysaccharide
MCP-1: monocyte chemoattractant protein 1
MRSA: methicillin-resistant *Streptococcus aureus*
NSCLC: nonsmall cell lung cancer
OH: hydroxyl ions
OOAC: organ-on-a-chip
PBMC: peripheral blood mononuclear cells
PDMS: polydimethylsiloxane
PLGA: poly(lactic-*co*-glycolic acid)
Poly(I:C): polyinosinic:polycytidylic acid
PM: particulate matter
PSCs: pluripotent stem cells
rhHGF: human hepatic growth factor
RNA: ribonucleic acid
ROS: reactive oxygen species
SARS: severe acute respiratory syndrome
SARS-CoV-2: severe acute respiratory syndrome coronavirus 2
TMPRSS2: transmembrane protease serine 2
TNF-α: tumour necrosis factor-α
TRPV4: transient receptor potential vanilloid 4
URTI: upper respiratory tract infection
VCAM-1: vascular cell adhesion molecule 1
WHO: World Health Organization
